# Increased prevalence of sex chromosome aneuploidies in specific language impairment and dyslexia

**DOI:** 10.1111/dmcn.12294

**Published:** 2013-10-09

**Authors:** Nuala H Simpson, Laura Addis, William M Brandler, Vicky Slonims, Ann Clark, Jocelynne Watson, Thomas S Scerri, Elizabeth R Hennessy, Patrick F Bolton, Gina Conti-Ramsden, Benjamin P Fairfax, Julian C Knight, John Stein, Joel B Talcott, Anne O'Hare, Gillian Baird, Silvia Paracchini, Simon E Fisher, Dianne F Newbury, SLI Consortium

**Affiliations:** 1 Wellcome Trust Centre for Human Genetics, University of OxfordOxford, UK; 2 Department of Clinical Neuroscience, Institute of Psychiatry, King's College LondonLondon, UK; 3 MRC Functional Genomics Unit, Department of Physiology, Anatomy & Genetics, University of OxfordOxford, UK; 4 Newcomen Centre, Evelina Children's HospitalLondon, UK; 5 Speech and Hearing Sciences, Queen Margaret UniversityEdinburgh, UK; 6 The Walter and Eliza Hall Institute of Medical ResearchMelbourne, Australia; 7 University Child Health and DMDE, University of AberdeenAberdeen, UK; 8 Departments of Child & Adolescent Psychiatry & Social Genetic & Developmental Psychiatry Centre, Institute of Psychiatry, King's College LondonLondon, UK; 9 School of Psychological Sciences, University of ManchesterManchester, UK; 10 University of OxfordOxford, UK; 11 Aston UniversityBirmingham, UK; 12 Department of Reproductive and Developmental Sciences, University of EdinburghEdinburgh, UK; 13 School of Medicine, University of St AndrewsSt Andrews, UK; 14 Max Planck Institute for PsycholinguisticsNijmegen, the Netherlands; 15 Donders Institute for Brain, Cognition and Behaviour, Radboud UniversityNijmegen, the Netherlands

## Abstract

**Aim:**

Sex chromosome aneuploidies increase the risk of spoken or written language disorders but individuals with specific language impairment (SLI) or dyslexia do not routinely undergo cytogenetic analysis. We assess the frequency of sex chromosome aneuploidies in individuals with language impairment or dyslexia.

**Method:**

Genome-wide single nucleotide polymorphism genotyping was performed in three sample sets: a clinical cohort of individuals with speech and language deficits (87 probands: 61 males, 26 females; age range 4 to 23 years), a replication cohort of individuals with SLI, from both clinical and epidemiological samples (209 probands: 139 males, 70 females; age range 4 to 17 years), and a set of individuals with dyslexia (314 probands: 224 males, 90 females; age range 7 to 18 years).

**Results:**

In the clinical language-impaired cohort, three abnormal karyotypic results were identified in probands (proband yield 3.4%). In the SLI replication cohort, six abnormalities were identified providing a consistent proband yield (2.9%). In the sample of individuals with dyslexia, two sex chromosome aneuploidies were found giving a lower proband yield of 0.6%. In total, two XYY, four XXY (Klinefelter syndrome), three XXX, one XO (Turner syndrome), and one unresolved karyotype were identified.

**Interpretation:**

The frequency of sex chromosome aneuploidies within each of the three cohorts was increased over the expected population frequency (approximately 0.25%) suggesting that genetic testing may prove worthwhile for individuals with language and literacy problems and normal non-verbal IQ. Early detection of these aneuploidies can provide information and direct the appropriate management for individuals.

## What this paper adds

- Individuals with language impairment have increased frequencies of sex chromosome aneuploidies.
- Routine screening of individuals with language and/or literacy problems should be considered.

Specific language impairment (SLI) is defined as a developmental language disorder that, in the absence of any comorbid neurological deficits, affects an individual's spoken and receptive language skills despite adequate intelligence and accessibility to learning. This common childhood disorder has an estimated prevalence in preschool children of up to 7%.^1^ It is a complex genetic disorder with a strong genetic background as found by family and twin studies.^2^

Dyslexia is a difficulty in reading and spelling that is unexpected, does not have an obvious cause,^3^ and occurs despite the individual having adequate instruction and intelligence. As with SLI, it is a common neurobehavioural disorder with a reported prevalence of 5% to 10% and strong evidence for a genetic contribution.^4^ Although they are considered distinct disorders, SLI has been shown to co-occur with dyslexia in some individuals,^5^ leading to the proposal that the two disorders share aetiological factors and contributory genetic variants.^6^ Common variations within the genes *CMIP* and *KIAA0319* have been implicated in both disorders.^7^,^8^

Sex chromosome aneuploidies, such as XXY, XYY, and XXX trisomies, occur within the general population at a rate of approximately 1 out of 400,^9^ and have been associated with a range of developmental and intellectual disabilities that often include language and reading problems.^10^ The rate of sex chromosome aneuploidies within children with specific developmental language disorders has been found to be as high as 5%.^11^ XXY (Klinefelter syndrome) and XYY occur within the male population with a prevalence of approximately 1 out of 600 and 1 out of 1000, respectively, and trisomy X occurs within the female population with a frequency of 1 out of 1000.^12^ It is of importance to identify sex chromosome aneuploidies at an early stage in an individual's life as a targeted intervention plan can then be implemented to help the individual.^13^

It is relatively common for a child with moderate to severe intellectual disability or an autism spectrum disorder (ASD) to undergo clinical karyotype or microarray analysis to identify causal cytogenetic or micro-duplication/deletion abnormalities. Karyotype analysis detects large chromosome rearrangements (3–10 Mb to whole chromosomes), whereas microarrays are used for smaller anomalies down to kilobase resolution. Either of these methods can be used to detect chromosome aneuploidies. The majority of cases of SLI and dyslexia are not routinely tested for genetic abnormalities. However, as language disorders may be associated with sex chromosome aneuploidies,^10^ we postulated that these traits could provide an early indication of sex chromosome abnormalities.

In this study we investigated the rates of sex chromosome aneuploidies in a clinical cohort of patients referred to a child development centre for speech and language problems. We then analysed a replication cohort of individuals with SLI, from both clinical and epidemiological samples and a sample set of individuals with dyslexia.

## Method

### Inclusion criteria for SLI group

The initial sample set analysed (language-impaired discovery cohort) included 87 probands and 165 siblings (252 individuals) recruited through the Newcomen Centre, Guys and St. Thomas' NHS Foundation Trust, London for a genetic study of SLI (the SLI Consortium [SLIC] study).^14^ Karyotype analysis was performed for all referrals as part of the clinical assessment, regardless of final diagnosis. This sample therefore contained some probands who ultimately were not included in the SLIC cohort as they did not meet the required criteria for the SLIC studies. The criteria for inclusion in SLIC were (1) a normal karyotype; and (2) language skills more than 1.5SD below that expected for their age on the Clinical Evaluation of Language Fundamentals^15^ expressive or receptive language scales; and (3) a non-verbal IQ above 80; and (4) no other obvious explanation for the impaired language, such as an ASD. Thirty-seven of the 87 probands (43%) in the clinical cohort did not meet these criteria and were excluded from the overall SLIC study. We had language data available for 30 of these 37 individuals. Of these, 19 (63%) had language ability more than 1.5 SD below that expected for their age but were excluded because of low non-verbal IQ (four cases) or the presence of a clinically diagnosed disorder which may explain their language difficulties (primarily ASD – 12 cases). In the present investigation, we chose to assess the entire sample as these individuals are representative of the population that may be referred with significant language impairment for potential investigation and they provide important information about the relationships between language impairment and other neurodevelopmental conditions.

Two-hundred and twelve independent language-impaired families (209 probands, three probands were not genotyped but these families contained affected siblings) and their siblings (187 individuals), were used as an SLI replication sample. SLI samples were recruited from clinical and epidemiological cohorts through the Cambridge Language and Speech Project (CLASP); the Child Life and Health Department at the University of Edinburgh; the Manchester language study and an independent case cohort from the Newcomen Centre, London. In contrast to the language-impaired cohort, all the replication SLI probands met the SLIC inclusion criteria described above with the exception of criterion (1) a normal karyotype, as the replication cohort were not karyotyped.

In both the discovery and replication cohorts DNA samples were also available for siblings and parents, and linguistic (Clinical Evaluation of Language Fundamentals) and psychometric data were collected for all children, regardless of language ability, to assess the level of language within family units. This allowed the identification of affected siblings, under the same criteria as that applied for the probands (described above, expressive language scale and/or receptive language scale >1.5SD below that expected and non-verbal IQ>80).

Given the high levels of co-occurrence, we did not exclude children affected by dyslexia from our language-impaired study samples. However, within these cohorts, reading and/or spelling data were available for 174 children with language impairment, allowing the estimation of the prevalence of concurrent reading impairment. Of the 174 children with data, 96 (55%) had reading or spelling scores more than 1SD below that expected for their age and 27 children (16%) had reading or spelling scores more than 2SD below that expected for their age.

### Inclusion criteria for dyslexia group

Three-hundred and fourteen unrelated children with dyslexia were selected from a dyslexia cohort recruited through the Dyslexia Research Centres in Oxford and Reading, and the Aston Dyslexia and Development clinic in Birmingham. The selected children had British Ability Scales-2 single-word reading scores of 100 or less (at chronological age) and more than 1.5SD below that predicted by their IQ scores.^7^ Probands were excluded if they self-reported the diagnosis of co-occurring developmental disorders such as SLI, autism, or attention-deficit–hyperactivity disorder.

### Comparison group

Two-hundred and eighty-seven healthy adult individuals (125 males, 162 females) were included as comparison participants. These individuals were originally recruited through local advertising in Oxfordshire for a study of gene expression in primary immune cells.^16^

Ethical permissions for each collection of samples were given by local ethics committees.

### Karyotyping

Karyotyping was only performed in the language-impaired cohort. Peripheral blood samples were collected from the language-impaired discovery (Newcomen Centre) cohort. Genomic DNA and chromosome suspensions were prepared according to standard procedures.^17^ Karyotype analysis at the 550–700 haploid band level was performed on G-banded chromosomes from the synchronized peripheral blood lymphocyte cultures of all individuals.

### Single nucleotide polymorphism (SNP) genotyping

**Figure 1:**
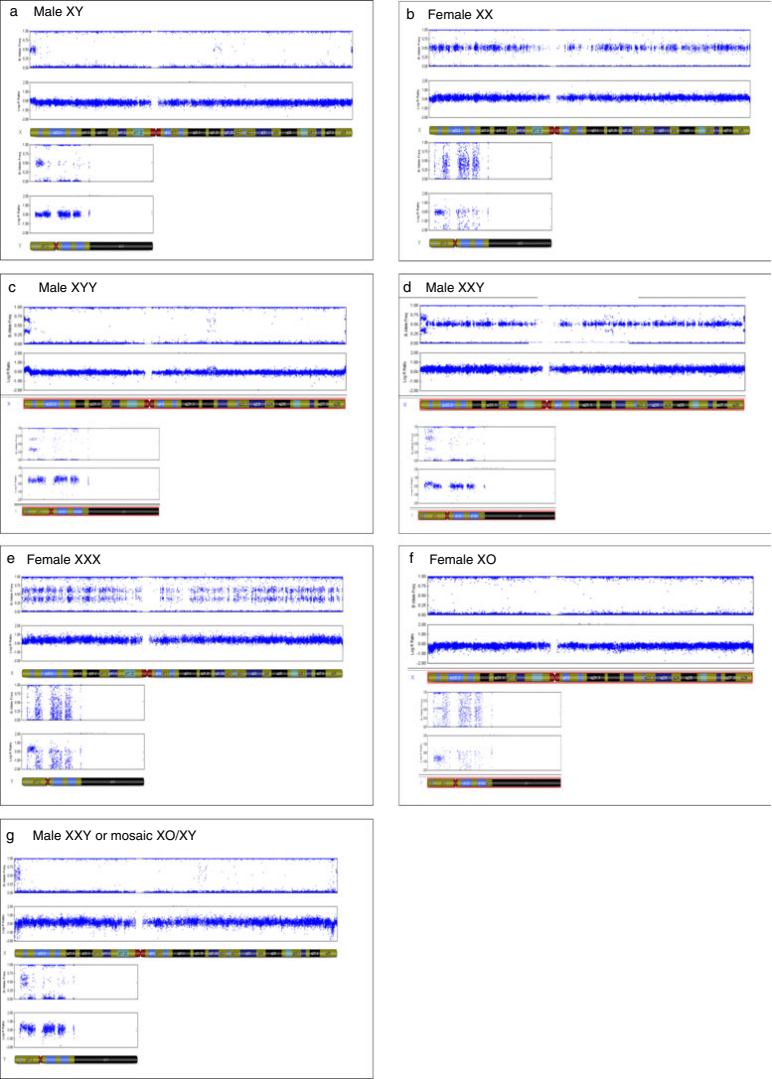
Examples of Genome Viewer (Illumina) outputs for individuals with and without sex chromosome aneuploidies. The plots display the B allele frequency (BAF) (top) and Log R ratio (LRR) (bottom) for each chromosome (X and Y) per individual. (a) and (b) are the expected XY and XX plots respectively. (c) XYY – Intermediates are seen for the BAF and an increase in LRR of the pseudoautosomal regions indicating an extra Y. (d) XXY – Heterozygote calls in the BAF and an increase in LRR across the entire X chromosome indicate two X chromosomes. Intermediates are also seen in the BAF in the pseudoautosomal regions (e) XXX – Intermediate BAF and an increase of LRR across the entire X indicate three X chromosomes. (f) XO – No heterozygote BAF calls and a decrease in LRR across the X chromosome signify a lack of an X chromosome. (g) XXY or XO/XY mosaic – Intermediate BAF in the pseudoautosomal regions and an increase of LRR across the entire X suggests either XXY with two identical X chromosomes or mosaicism with loss of the Y chromosome (Figure on next page).

In those cases where karyotypes were not available, sex chromosome aneuploidies were identified using genomewide SNP arrays. DNA samples were extracted from peripheral blood or buccal smears for all individuals in the SLI replication, dyslexia, and control cohorts. These, and 54 individuals from the discovery sample (who had also been karyotyped and therefore provided positive controls), were genotyped on the Illumina HumanOmniExpress-12v1 Beadchip (Illumina Inc., San Diego, CA, USA) that contains approximately 750 000 SNPs. SNP data were subjected to principal components analysis in Eigenstrat^18^ to assess the ethnicity of all samples against HapMap3 control samples.^19^ Thirteen individuals from the language-impaired discovery cohort, 21 from the SLI replication cohort, 11 from the dyslexia cohort, and 15 comparison participants were found to have some non-European ancestry. As this factor was not thought to increase the chance of sex chromosome aneuploidies, these individuals were left in the sample sets. In total, 18 159 X chromosome SNPs, 1679 Y chromosome SNPs, and 572 pseudoautosomal SNPs were analysed. Log R ratio and B allele frequency plots were visually inspected within GenomeStudio Software (Illumina) for loss or gain of X and Y chromosomes. Figure1 shows examples of Log R ratio and B allele frequency plots for XX, XY, XYY, XXY, XXX, and XO. Two-tailed Fisher's exact tests were performed for the number of sex chromosome aneuploidies in each cohort.

### DNA polymorphism genotyping

Thirteen polymorphisms on the X and Y chromosomes^20^,^21^ were genotyped in the 12 individuals identified as having sex chromosome abnormalities and their available family members. This process served to validate the aneuploidies detected and allowed the designation of parental origin and mechanism for several cases. Polymerase chain reaction products were pooled in a ratio of 1:1 per individual, diluted 1 in 20 and 2μl loaded onto an Applied Biosystems 3730xl analyzer (Life Technologies, Foster City, CA, USA). Genotypes were annotated using Peak Scanner Software (v1.0; Applied Biosystems, Foster City, CA, USA). Some polymorphisms were X or Y chromosome specific, whereas others lay in the pseudoautosomal regions.

## Results

In total, two XYY, four XXY (Klinefelter syndrome), three XXX, one XO (Turner syndrome), and one unresolved XXY or XO/XY mosaic karyotype were identified in probands and one XYY in an affected sibling (TableI). An increased prevalence of sex chromosome aneuploidies was found across all three populations studied suggesting that genetic testing may prove worthwhile for individuals with severe speech and language and/or literacy problems.

**Table I:** Percentage of each aneuploidy in each cohort according to sex. (*n* of individuals)

	Total	Males:Females, *n*	XYY	XXY	XXX	XO	XXY or XO/XY mosaic
LI discovery probands	87	61:26	3.3 (2)	0	3.8 (1)	0	0
LI discovery siblings	165	102:63	1.0 (1)	0	0	0	0
SLI replication probands	209	139:70	0	2.2 (3)	2.9 (2)	0	0.7(1)
SLI replication siblings	187	99:88	0	0	0	0	0
Dyslexia	314	224:90	0	0.4 (1)	0	1.1 (1)	0
Control participants	287	125:162	0	0	0	0	0
Expected frequency from literature			0.1	0.17	0.1	0.05	0.007 (XO/XY mosaic)

LI, language impairment; SLI, specific language impairment.

### Language-impaired discovery cohort

Karyotypic analyses were performed for all probands in the language-impaired discovery cohort and their siblings, regardless of affection status, and consisted of 252 children from 87 families (87 probands [61 males, 26 females] and 165 siblings [102 males, 63 females]).

In this discovery cohort, four abnormal karyotypic results were identified representing a population yield of 1.58%. These individuals included three probands (proband yield, 3.4%) and one sibling who had language and developmental concerns (sibling yield, 0.6%). This is at a much-increased rate compared with the general population frequency (approximately 0.25%)^9^ and, in the probands, is a significant increase over that observed in our comparison participant samples (*p*=0.012).

Of these four individuals, three (two probands and one independent sibling) were XYY (individuals LI _1, LI_2 and LI_3 in TableII respectively), and one had Triple X syndrome (XXX) (LI_10 in Table2).

**Table II:** The sex chromosome aneuploidies identified

Individual	Sex chromosome aneuploidy	Disorder	Sex	Confirmed by karyotyping	Causative mechanism	Maternal age at birth	Paternal age at birth
LI_1 proband	XYY	SLI	Male	Yes	Paternal MII non-disjunction	33	39
LI_2 proband	XYY	SLI	Male	Yes	Paternal MII non-disjunction	32	32
LI_3 affected sib	XYY	SLI	Male	Yes	Paternal MII non-disjunction	37	33
SLI_4 proband	XXY	SLI	Male	ND	Maternal MII non-disjunction	34	35
SLI_5 proband	XXY	SLI	Male	ND	Maternal MII non-disjunction	NA	NA
SLI_6 proband	XXY	SLI	Male	ND	Paternal MI non-disjunction	NA	NA
DYS_7 proband	XXY	Dyslexia	Male	ND	NA	NA	NA
SLI_8 proband	XXX	SLI	Female	ND	Maternal MII non-disjunction	36	36
SLI_9 proband	XXX	SLI	Female	ND	Maternal MI non- disjunction	34	39
LI_10 proband	XXX	SLI	Female	Yes	Paternal MII non-disjunction	33	35
DYS_11 proband	XO	Dyslexia	Female	ND	NA	NA	NA
SLI_12 proband	XXY or XO/XY mosaicism	SLI	Male	ND	NA	26	26

LI, language impairment; SLI, specific language impairment; MII, meiosis II; ND, not done; NA, not available; MI, meiosis I.

The karyotypic anomalies were supported by two independent methodologies: visual inspection of SNP genotype data in the form of Log R ratio and B allele frequency plots (Fig.1), and the investigation of a panel of X and Y chromosome DNA polymorphisms.

Two of the XYY males were in special needs schools and one was in a mainstream school with a statement of special educational needs. All three XYY males would have met SLIC criteria for SLI (as specified in the Method section) if not for their chromosomal abnormalities. One had severe language delay and the other two had additional articulation problems. All were receiving speech and language therapy.

The XXX female proband had articulation difficulties without receptive language delay and was receiving speech and language therapy at a language unit attached to a mainstream school. At the time of ascertainment she was too young for formal assessment.

As noted in the Method section, the probands in the discovery cohort were a self-referred sample of children with persistent speech and language problems, needing special schooling and thus may not be considered representative of the total population in the community. We, therefore, subsequently investigated a replication cohort in which all probands were selected to meet diagnostic criteria for SLI before sex chromosome investigation.

### SLI replication cohort

A further 212 families containing an individual with SLI were scrutinized for abnormalities using SNP data from the Illumina HumanOmniExpress beadchip (see Fig.1 for examples of how the abnormalities were visualized). This included 209 probands (139 male, 70 female [three probands were not genotyped but these families included an affected sibling]) and 187 siblings (99 males, 88 females). Importantly, these individuals were collected through population-based samples as well as clinical cohorts and all probands met diagnostic criteria for SLI.

In this replication cohort, six sex chromosome abnormalities were identified representing a population yield of 1.5%, consistent with that observed in the discovery cohort, and statistically significant when compared with the comparison cohort (*p*=0.005). All six abnormalities occurred in probands (proband yield, 2.9%). These findings contrast with the sibling and control data, in which no aneuploidy of the sex chromosomes was found. Three male probands (SLI_4, SLI_5, and SLI_6) had Klinefelter syndrome (XXY), two female probands had XXX (SLI_8 and SLI_9), and one male proband had an unusual SNP data pattern, which may be interpreted as either XXY with two identical maternal X chromosomes or an XO/XY mosaic (SLI_12; see Fig.1, Table2). As they matured, all three males with Klinefelter syndrome exhibited complex developmental issues surrounding their speech and language difficulties. SLI_5 (XXY) was reported as being anxious and SLI_6 (XXY) had comorbid hyperactivity. SLI_4 was very tall for his age and had been identified as having Klinefelter syndrome since being recruited.

### Dyslexia samples

Given the increased number of sex chromosome aneuploidies in the language-impaired and SLI cohorts, we wanted to ascertain if this also occurred in individuals with the written language disorder dyslexia. A total of 314 independent cases were investigated and two were found to have sex chromosome aneuploidies representing a proband yield of 0.6%. Although higher than expected in the general population (approximately 0.25%),^9^ this is not statistically significant when compared with the comparison cohort (*p*=0.502). One male (DYS_7) had Klinefelter syndrome (XXY) and one female (DYS_11) had Turner syndrome (XO) (Table2). The proband with Turner syndrome had severe reading difficulties with reading and spelling scores more than 1.5SD below that expected for her age. The proband with Klinefelter syndrome was less severely affected but still scored approximately 0.5SD below that expected for his age on both reading and spelling tests. Clinical information was not available for the individuals with dyslexia but we understand that a diagnosis of Turner syndrome had been made for the female proband.

### Comparison samples

In contrast with the language-impaired and dyslexic samples, no aneuploidies were identified in the 287 comparison individuals investigated.

### Mechanism of aneuploidies

Genotyping of polymorphisms across the X and Y chromosomes allowed the derivation of a putative mechanism of abnormality in 10 of the 12 cases. Five abnormalities were paternal in origin, four were maternal, and one was post-zygotic (Fig. S1, online supplementary information.).

## Discussion

In this study, we investigated the prevalence of sex chromosome aneuploidies within individuals with language and reading problems, the majority of whom do not have other developmental delays.

Karyotype and SNP genotyping analyses of 87 and 209 probands with language impairment from the discovery and replication cohorts respectively, and 314 individuals with dyslexia identified 11 sex chromosome aneuploidies. One additional abnormality was also identified in an affected sibling, who also had speech and language concerns. The proband frequency of aneuploidies was 3.4%, 2.9%, and 0.6% in the language-impaired discovery, SLI replication, and dyslexia cohorts respectively. Importantly, the frequency of aneuploidies found in the language-impaired siblings (0.28%) was in line with the expected population frequency (0.25%)^9^ and no aneuploidies were discovered in the comparison (*n*=287) or SLI parental (*n*=429) samples investigated. Probands with oral speech and language deficits showed a higher yield than those with developmental dyslexia (3.4% and 2.9% vs 0.6% respectively). This elevated prevalence is in line with that of 5% reported previously.^11^ Accordingly, the increased prevalence in both language-impaired cohorts was statistically significant when compared with the control cohort but the dyslexia cohort was not. Similarly, the rate of each individual aneuploidy (Table1) within the separate cohorts when analysed by sex was not significantly increased over that of the controls. This is likely to be the result of small sample sizes when the sample is decomposed in this way.

Although most adults with sex chromosome trisomies live independent lives, they often have poor verbal ability and experience behavioural and social difficulties.^10^ Males with XYY have an increased risk of developmental delays and ASD.^22^ The XYY males in our language-impaired discovery cohort all had language difficulties, developmental delays, and special educational needs, although this may represent an ascertainment bias of this clinically selected cohort. Nonetheless, a similarly increased frequency of sex chromosome aneuploidies was observed in the replication cohort which included both clinically and epidemiologically selected individuals with SLI, indicating that the observed frequencies extend across the whole language-impairment spectrum. Furthermore, the discovery of two sex chromosome aneuploidies in the dyslexia cohort suggests that sex chromosome abnormalities may also be associated with reading difficulties in the absence of overt oral language problems.

Many of the individuals studied here had not been clinically karyotyped and were perhaps unaware of their diagnosis. Although some symptoms of sex trisomies may be detected during development leading to a clinical referral for karyotyping, it is suggested that as many as 90% of cases go undetected.^23^ If detected at an earlier stage a targeted intervention plan could be implemented to help the individual and the family to plan for lifetime impact of the disorder.^13^

The most likely genetic cause of the language and reading problems within individuals with X chromosome aneuploidies is the altered dosage of genes in the telomeric pseudoautosomal regions of the X and Y chromosomes. The pseudoautosomal regions on the X and Y chromosomes are identical and escape X inactivation. Thus there would be an increase in gene dosage for trisomic individuals and a decrease for monosomic individuals, which presumably would be detrimental. Similarly, individuals with Y chromosome aneuploidies will possess an altered gene dosage for genes on the Y chromosome.

One individual appeared to have a normal male SNP pattern for the X and Y chromosomes but nonetheless showed a split signal across the pseudoautosomal regions indicating the presence of extra genetic material (Fig.1). The most likely interpretation of this pattern would be an XXY karyotype with two identical maternal X chromosomes. However, a similar pattern would be expected for an XO/XY mosaic karyotype. This is a rare condition (population frequency less than 1 out of 15 000 live births) that does not always have a distinctive phenotype.^24^ Resolution of the exact karyotype is not possible in this case as the DNA sample was obtained from a buccal swab and no follow-up is available.

The majority of XXX and XXY cases in the general population are maternal in origin,^25^ because of the length of time that meiosis I lasts in females. This is consistent with our data, where four of the six XXX and XXY cases, in whom the origin could be determined, were maternal.

With ever increasing numbers of DNA samples being analysed on SNP genotyping microarrays, it has become easier to identify sex chromosome aneuploidies. Our data indicate that microarray traces are sensitive to and sufficient for the detection of these abnormalities. However, it should be noted that many of our cases were not identified by standard quality control procedures or picked up by copy number variant algorithms, which look for local variations in signal intensities and allele frequencies. Thus, the identification of these cases, particularly XYY and certain instances of XXY as discussed above, demands the visual inspection of SNP data within the appropriate software, which is time consuming and perhaps may not be feasible in a clinical setting.

The increased frequency of sex chromosome aneuploidies within language- and reading-impaired individuals found in this study indicates that karyotype testing or microarray examination would be useful for children with severe language impairment even if their non-verbal IQ is within the normal range, as they may be missed by current clinical screening. To our knowledge, the majority of the sex chromosome aneuploidies detected had not previously been diagnosed. However, it should be noted that, by definition, all the affected individuals investigated in this paper had severe language and/or reading problems. Further studies of alternative cohorts will be required to accurately assess the frequency of sex chromosome abnormalities across different severities and alternative behavioural disorders. As the phenotypes of speech and language delay appear in childhood, this could be an indicator of sex chromosome abnormalities before specific physical phenotypes appear. Early detection can provide information about the problems that may be associated with these disorders and appropriate management.^13^ Therefore, karyotyping should be considered for individuals with language and/or reading problems upon diagnosis.

## Abbreviations

ASD: Autism spectrum disorder
SLI: Specific language impairment
SLIC: Specific Language Impairment Consortium
SNP: Single nucleotide polymorphism

## Supporting Information

**Figure S1:** Causative mechanisms of the sex chromosome aneuploidies.
