# Supplementary material for: Increased prevalence of sex chromosome aneuploidies in specific language impairment and dyslexia
Source: Dev Med Child Neurol. 2013 Oct 9;56(4):346–53. doi: 10.1111/dmcn.12294 (PMC4293460; doi:10.1111/dmcn.12294)
Supplement: Supplementary file 1 — Figure S1: Causative mechanisms of the sex chromosome aneuploidies. [file dmcn0056-0346-sd1.pdf]

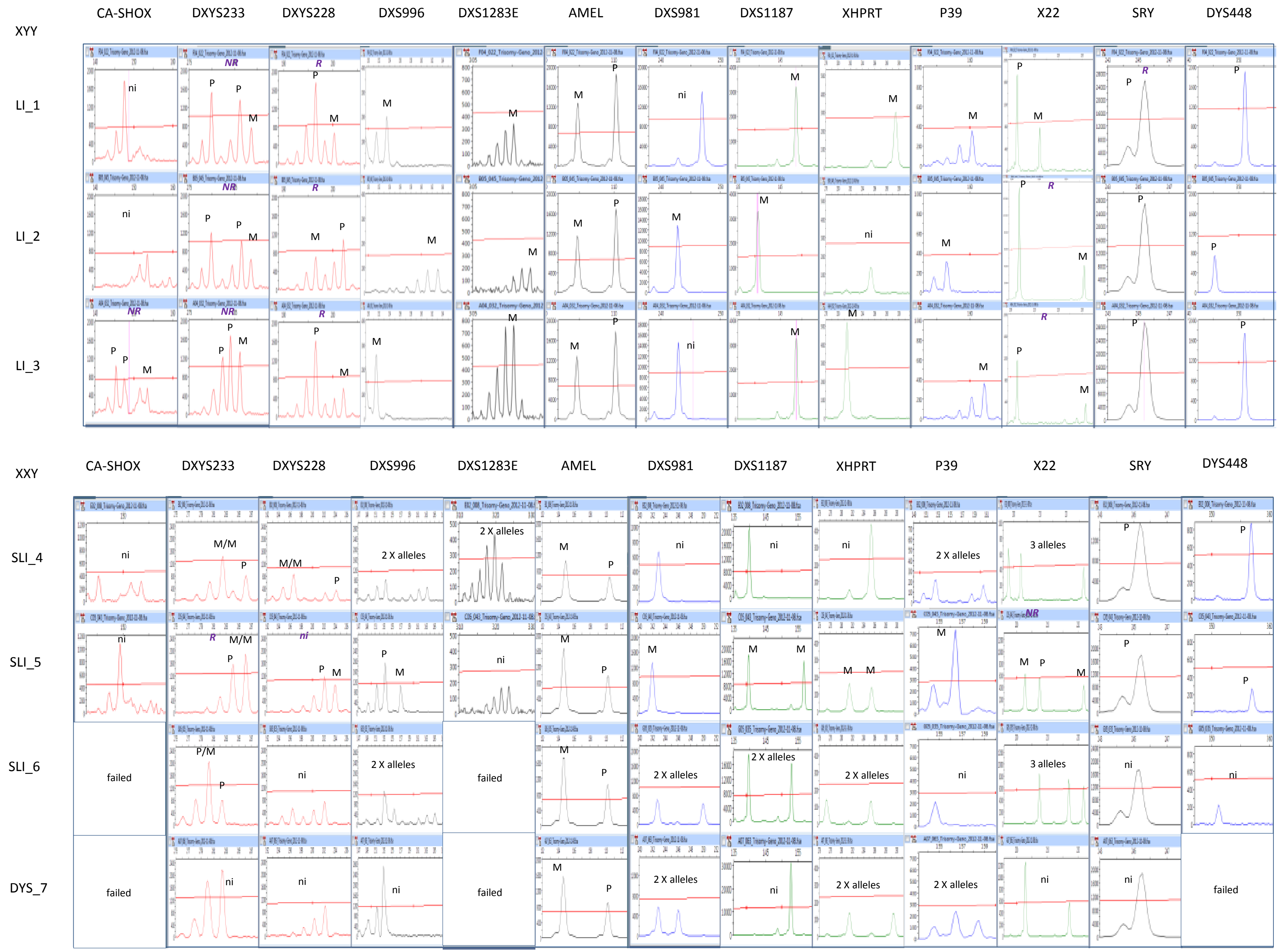
XYY
CA-SHOX
DXYS233
DXYS228
DXS996
DXS1283E
AMEL
DXS981
DXS1187
XHPRT
P39
X22
SRY
DYS448
LI_1
LI_2
LI_3
XYY
CA-SHOX
DXYS233
DXYS228
DXS996
DXS1283E
AMEL
DXS981
DXS1187
XHPRT
P39
X22
SRY
DYS448
SLI_4
SLI_5
SLI_6
DYS_7

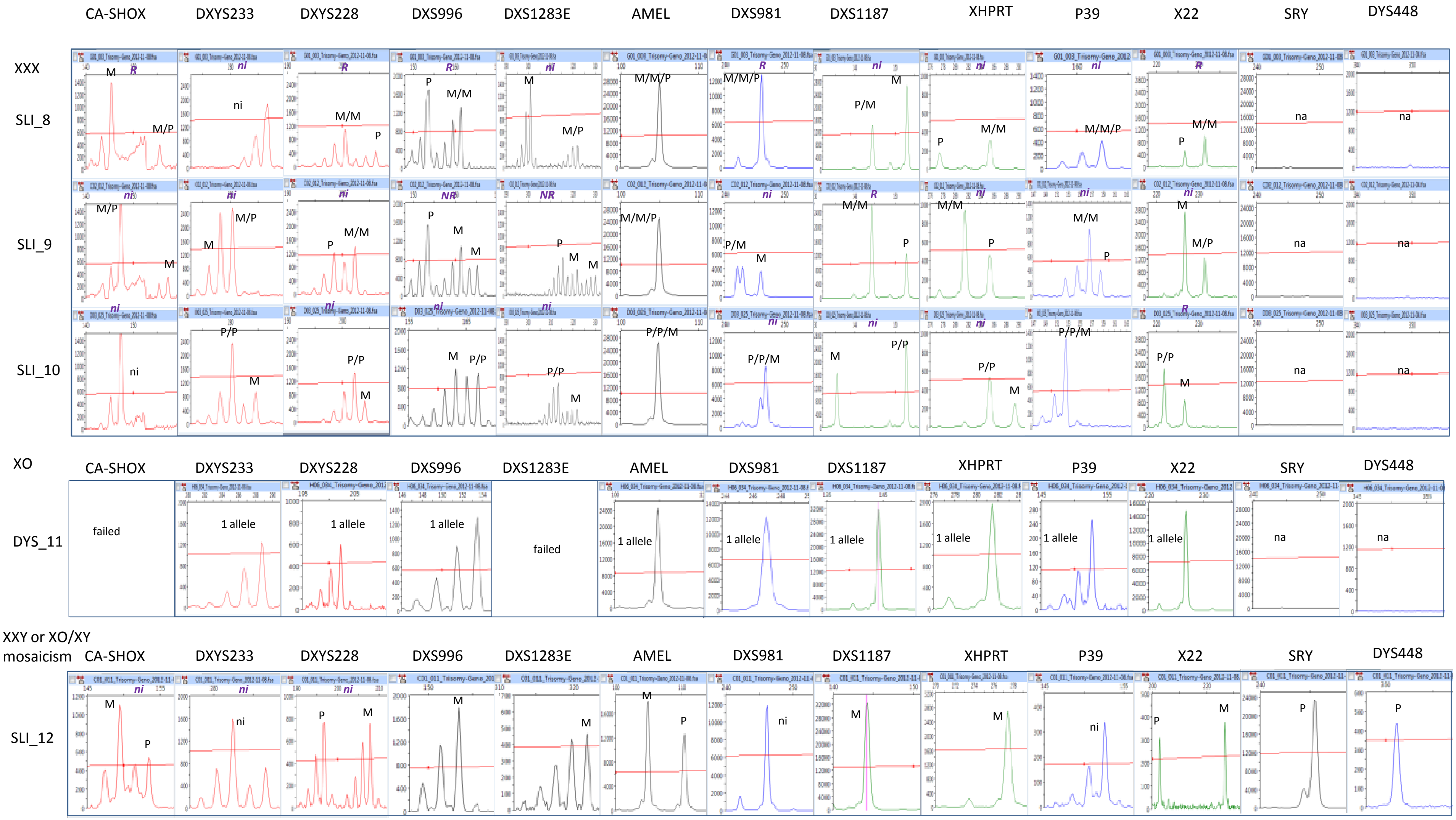

R heterozygosity in the parent reduced to homozygosity in the offspring
NR heterozygosity in the parent not reduced to homozygosity in the offspring
ni non-informative
na not applicable
